# Automated segmentation of thoracic aortic lumen and vessel wall on three-dimensional bright- and black-blood magnetic resonance imaging using nnU-Net

**DOI:** 10.1016/j.jocmr.2025.101923

**Published:** 2025-06-11

**Authors:** Matteo Cesario, Simon J. Littlewood, James Nadel, Thomas J. Fletcher, Anastasia Fotaki, Carlos Castillo-Passi, Reza Hajhosseiny, Jim Pouliopoulos, Andrew Jabbour, Ruperto Olivero, Jose Rodríguez-Palomares, M. Eline Kooi, Claudia Prieto, René M. Botnar

**Affiliations:** a School of Biomedical Engineering and Imaging Sciences, King’s College London, London, United Kingdom; b Department of Radiology and Nuclear Medicine, Maastricht University Medical Centre, Maastricht, the Netherlands; c Cardiovascular Research Institute Maastricht (CARIM), Maastricht University, Maastricht, the Netherlands; d Clinical Research Group, Heart Research Institute, Newtown, Australia; e Cardiology Department, St. Vincent’s Hospital, Darlinghurst, Australia; f Institute of Biological and Medical Engineering, Pontificia Universidad Católica de Chile, Santiago, Chile; g Department of Cardiology, Chelsea and Westminster Hospital NHS Foundation Trust, London, United Kingdom; h Victor Chang Cardiac Research Institute, Sydney, Australia; i Department of Cardiology, Vall d′Hebron Hospital Universitari, Vall d′Hebron Barcelona Hospital Campus, Barcelona, Spain; j Department of Medicine, Universitat Autònoma de Barcelona, Bellaterra, Spain; k Cardiovascular Diseases, Vall d′Hebron Institut de Recerca (VHIR), Vall d′Hebron Barcelona Hospital Campus, Universitat Autònoma de Barcelona, Bellaterra, Spain; l CIBER de Enfermedades Cardiovasculares, Instituto de Salud Carlos III, Madrid, Spain; m School of Engineering, Pontificia Universidad Católica de Chile, Santiago, Chile; n Millennium Institute for Intelligent Healthcare Engineering, Santiago, Chile; o Institute for Advanced Study, Technical University of Munich, Garching, Germany

**Keywords:** Aorta, Aortic disease, Magnetic resonance angiography, Segmentation, Deep-learning, nnUNet

## Abstract

**Background:**

Magnetic resonance angiography (MRA) is an important tool for aortic assessment in several cardiovascular diseases. Assessment of MRA images relies on manual segmentation, a time-intensive process that is subject to operator variability. We aimed to optimize and validate two deep-learning models for automatic segmentation of the aortic lumen and vessel wall in high-resolution electrocardiogram-triggered free-breathing respiratory motion-corrected three-dimensional (3D) bright- and black-blood MRA images.

**Methods:**

Manual segmentation, serving as the ground truth, was performed on 25 bright-blood and 15 black-blood 3D MRA image sets acquired with the iT2PrepIR-BOOST sequence (1.5T) in thoracic aortopathy patients. The training was performed with no new U-Net (nnUNet) for bright-blood (lumen) and black-blood image sets (lumen and vessel wall). Training consisted of a 70:20:10% (17/25:5/25:3/25 datasets) training:validation:testing split. Inference was run on datasets (single vendor) from different centers (UK, Spain, and Australia), sequences (iT2PrepIR-BOOST, T2 prepared coronary magnetic resonance angiography [CMRA], and time-resolved angiography with interleaved stochastic trajectories [TWIST] MRA), acquired resolutions (from 0.9–3 mm^3^), and field strengths (0.55T, 1.5T, and 3T). Predictive measurements comprised Dice similarity coefficient (DSC) and Intersection over Union (IoU). Postprocessing (3D slicer) included centreline extraction, diameter measurement, and curved planar reformatting (CPR).

**Results:**

The optimal configuration was the 3D U-Net. Bright-blood segmentation at 1.5T on iT2PrepIR-BOOST datasets (1.3 and 1.8 mm^3^) and 3D CMRA datasets (0.9 mm^3^) resulted in DSC ≥ 0.96 and IoU ≥ 0.92. For bright-blood segmentation on 3D CMRA at 0.55T, the nnUNet achieved DSC and IoU scores of 0.93 and 0.88 at 1.5 mm³, and 0.68 and 0.52 at 3.0 mm³, respectively. DSC and IoU scores of 0.89 and 0.82 were obtained for CMRA image sets (1 mm^3^) at 1.5T (Barcelona dataset). DSC and IoU scores of the BRnnUNet model were 0.90 and 0.82, respectively, for the contrast-enhanced dataset (TWIST MRA). Lumen segmentation on black-blood 1.5T iT2PrepIR-BOOST image sets achieved DSC ≥ 0.95 and IoU ≥ 0.90, and vessel wall segmentation resulted in DSC ≥ 0.80 and IoU ≥ 0.67. Automated centreline tracking, diameter measurement, and CPR were successfully implemented in all subjects.

**Conclusion:**

Automated aortic lumen and wall segmentation on 3D bright- and black-blood image sets demonstrated excellent agreement with ground truth. This technique demonstrates a fast and comprehensive assessment of aortic morphology with great potential for future clinical application in various cardiovascular diseases.

## Graphical abstract

**Figure:**
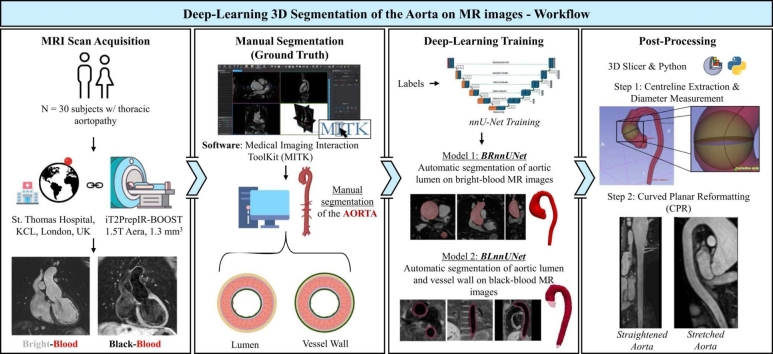
Figure partially created in BioRender. Cesario, M. (2025) https:// BioRender.com/7cpyxat. Additional icons sourced from Flaticon under Premium license.

## 1 Introduction

Aortic diseases, known as aortopathies, represent a spectrum of cardiovascular (CV) disorders characterized by structural abnormalities of the aorta, predisposing individuals to the development of aneurysms and acute aortic syndromes, such as dissections 1, 2, 3. Aortic aneurysms (AAs) are broadly classified as thoracic (TAA) or abdominal and are characterized by a localized increase in diameter of ≥50% when compared to sex and age-adjusted healthy individuals 1, 2, 3. Clinical symptoms of AAs typically manifest as dissection or rupture, which are fatal in most circumstances 1, 2, and make up the majority of acute aortic syndromes, which also include intramural hematoma, penetrating atherosclerotic ulcers, and traumatic aortic injury. Precise diagnostic imaging for the assessment and surveillance of aortic diseases is essential 1, 2.

In recent years, vascular magnetic resonance imaging (MRI) has become an important imaging modality for the assessment of aortic disease [4]. This is largely due to its lack of ionizing radiation, high sensitivity and specificity, and superior soft tissue contrast compared to other imaging techniques (i.e., computed tomography angiography [CTA] and transthoracic echocardiography) [4]. One of the key parameters for aortic disease remains the assessment of aortic diameter 5, 6, although vessel wall thickness also has important clinical implications [4]. Bright- and black-blood (BR and BL) magnetic resonance (MR) sequences are included in clinical guidelines for the visualization of the aortic lumen (BR signal) and vessel wall (nulled blood signal = BL signal), respectively 4, 7. Conventionally, these sequences are acquired separately, limiting acquisition efficiency, increasing scan times, and often resulting in misregistration artifacts 4, 7. The novel MR sequenceinterleaved T2 and inversion recovery prepared bright-blood and black-blood phase-sensitive sequence (iT2PrepIR-BOOST) allows simultaneous, co-registered acquisition of BR and BL images with reduced scan times and improved contrast compared to standard non-contrast-enhanced (non-CE) BR three-dimensional (3D) magnetic resonance angiography (MRA) and two-dimensional (2D) breath-hold BL half-Fourier acquisition single-shot turbo spin echo imaging sequences, respectively, and has been recently demonstrated in patients with aortopathies 4, 7.

Measurement of aortic diameter and vessel wall thickness relies on manual segmentation of MRI data 8, 9, 10, which is labor-intensive, time-consuming, and subject to intra- and inter-rater variability and expertise, especially on large 3D datasets 4, 10. Automatization of this process through the development of deep-learning (DL) models could enhance diagnostic processes and workflow by reducing reliance on manual segmentation, minimizing operator dependence, increasing reproducibility [10], and facilitating more timely and accurate clinical decisions, ultimately improving patient outcomes 10, 11. Most of the available literature on automatic aortic lumen segmentation focuses on CTA image sets with the use of U-Nets 11, 12, 13, with a smaller percentage focusing on 3D MRI sequences and four-dimensional (4D) flow acquisitions 14, 15, 16, 17. The “nnU-Net” is an open-source extension of 2D and 3D U-Nets and includes automated hyperparameter and model selection for optimal image segmentation [18]. The nnUNet was proven to have superior performance compared to other automatic segmentation methods 18, 19 and its application has been shown for a wide range of CV MRI tasks 20, 21. To further aid generalizability, it is important to create a robust model that has been tested and trained on datasets across different cardiovascular magnetic resonance (CMR) scanners, sequences, acquired resolutions, and centers [22].

In this study, we sought to optimize and evaluate two nnUNet models for the automatic segmentation of the aortic lumen and vessel wall on 3D MRI acquired using iT2PrepIR-BOOST 4, 7. The DL model is referred to as *BRnnUNet* (BR nnUNet model trained to segment aortic lumen) for the non-CE BR image sets (lumen segmentation) and *BLnnUNet* for the BL image sets (combined vessel wall and lumen segmentation). Both models allow the output of 3D contours and 3D aortic rendering. To evaluate generalizability, the networks are evaluated during inference on datasets with different contrasts, acquired resolutions, MR scanner models, and field strengths for a single vendor. Open-source software was then employed to automate the tracking of the vessel’s centreline from the DL-generated 3D volumes, to facilitate fast and accurate measurements of aortic diameters and cross-sectional area (CSA), and automatically perform curved planar reformatting (CPR) to facilitate viewing in a single plane.

## 2 Materials and methods

**Fig. 1:**
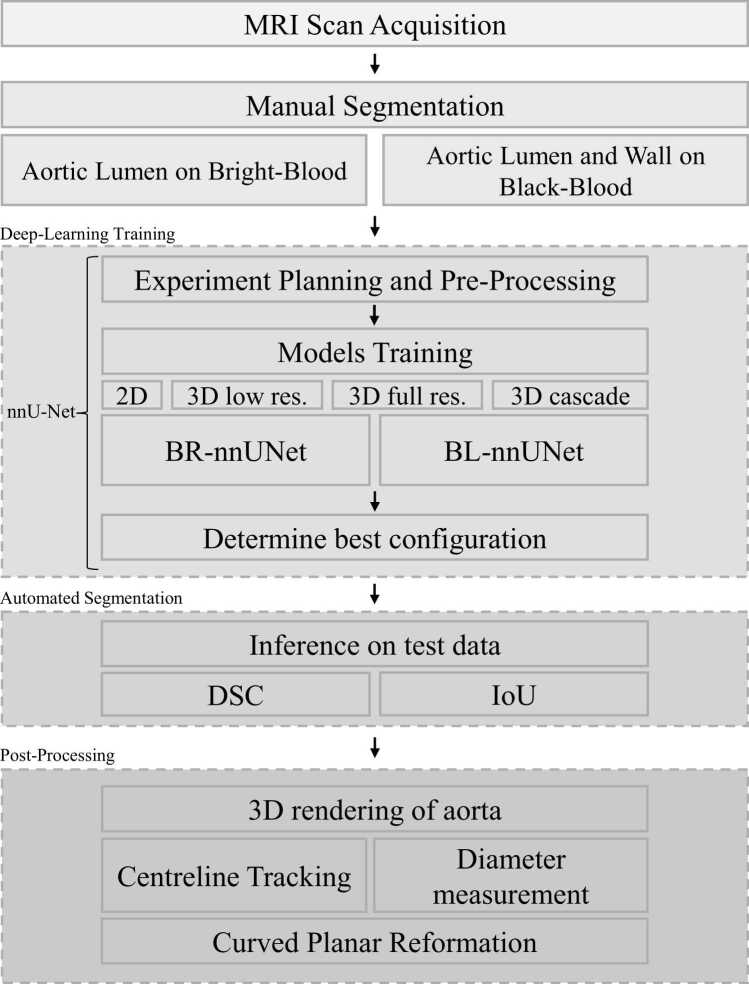
Projec workflow. The study consisted of the acquisition of N = 30 MRI scans with the iT2PrepIR-BOOST sequence, which were then manually segmented to produce labels/annotations for deep-learning model training. The latter was performed with nnUNet. The two models—*BRnnUNet* and *BLnnUNet*—were tested on unseen test data, and predictive measures (DSC and IoU) were calculated. Postprocessing of the 3D masks consisted of (automatic) centerline tracking, diameter measurement, and curved planar reformatting. *2D* two-dimensional, *3D* three-dimensional, *BRnnUNet* bright-blood nnUNet model trained to segment aortic lumen, *BLnnUNet* black-blood nnUNet model trained to segment aortic lumen and wall, *DSC* Dice similarity coefficient, *IoU* Intersection over Union, *MRI* magnetic resonance imaging, *nnUNet* no new U-Net

An outline of the study’s workflow is shown in Fig. 1. The iT2PrepIR-BOOST 4, 7 MR image acquisition was followed by manual segmentation to generate ground truth (GT) labels for training. DL training was performed with nnUNet [18], followed by inference on various testing datasets that had different acquired spatial resolutions, acquired with different MR scanners, field strengths, and pre-pulses (IR-T2-Prep vs T2-Prep). Finally, postprocessing consisted of (automatic) centreline extraction, diameter measurement, and CPR using open-source software.

### 2.1 Ethical approval

This study was performed in accordance with the Declaration of Helsinki and approved by the National Research Ethics Service (REC 15/NS/0030). Written informed consent was obtained from each participant according to institutional guidelines.

### 2.2 MRI training data acquisition

#### 2.2.1 Study population

Image sets used for DL training (N = 30) were obtained from the study by Munoz et al. [4]. Briefly, non-CE BR and BL datasets were acquired using the 3D iT2PrepIR-BOOST sequence from 30 individuals with thoracic aortopathies (male = 21; age = 32 ± 12 years; height = 178 ± 12 cm; weight = 78 ± 13 kg). Aortic conditions ranged from AAs to aortic coarctation, including aortic root dilatation, aortic compression, and bicuspid aortic valves, among others (Supplementary Fig. S1).

#### 2.2.2 MR sequence(s) parameters

The iT2PrepIR-BOOST sequence 4, 7 allows free-breathing, co-registered and motion-corrected acquisition of 3D BR and BL image sets in a short scan time of 7.8 ± 1.7 min 4, 7. It employs an electrocardiogram-triggered balanced steady state free precession, preceded by a T2 preparation (T2-Prep) (duration = 40 ms) and inversion recovery (IR) (inversion time = 110 ms) pre-pulse (iT2PrepIR) in odd heartbeats, and chemical shift-based fat saturation in even heartbeats 4, 7. The sequence generates two sets of non-CE BR image volumes. The first volume can be used for BR visualization of the heart and major blood vessels (lumen visualization), while the second (reference) dataset can be used to obtain a T1-weighted BL image volume by magnitude subtraction of the odd from the even heartbeat dataset 4, 7. The iT2PrepIR-BOOST was acquired on a 1.5T MR scanner (MAGNETOM Aera, Siemens Healthcare, Erlangen, Germany), at an isotropic acquired resolution of 1.3 mm^3^ [4]. Information about the image acquisition parameters, including the use of image navigators (iNAVs) for respiratory motion correction and multi-contrast patch-based low-rank denoising (HD-PROST) [23] has been previously described [4].

### 2.3 MRI testing data acquisition

Testing data (N = 31) was acquired at three different sites: St. Thomas Hospital (London, UK), Vall d′Hebron University Hospital (Barcelona, Spain), and St. Vincent Hospital (Sydney, Australia). Data were acquired at 0.55T (Free MAX, Siemens Healthcare, Erlangen, Germany), 1.5T (Aera and Sola, Siemens Healthcare, Erlangen, Germany), and 3T (Prisma, Siemens Healthcare, Erlangen, Germany) scanners with acquired resolutions ranging from 0.9 to 3 mm^3^. Acquisitions were performed with iT2PrepIR-BOOST, with similar parameters to the training dataset, except for variations in acquired resolution. Ten datasets acquired using T2-Prep coronary magnetic resonance angiography (CMRA) with iNAV and HD-PROST reconstruction for BR imaging only were included in the test data [24]. Scan parameters for the CMRA technique can be found in the original article [24]. One contrast-enhanced (CE) dataset was acquired with the “Time-resolved angiography With Interleaved Stochastic Trajectories” (TWIST) MRA sequence [25].

A summary of the training and testing data is shown in Table 1.

**Table 1:** MR sequence specifics for the datasets employed in the study.

Center (city, country)	Sequence	Scanner	Acquired resolution	Image	N	Used for	Aortic disease (Y/N/Mix)
St. Thomas Hospital, KCL (London, UK)	iT2PrepIR-BOOST	1.5T Aera	1.3 mm³	BR	18/4/3	Training/validation/testing	Y
				BL	11/4/8		
St. Thomas Hospital, KCL (London, UK)	iT2PrepIR-BOOST	1.5T Sola	1.8 mm³	BR	4	Testing	N
				BL	2		
St. Thomas Hospital, KCL (London, UK)	CMRA (T2-Prep)	1.5T Sola	0.9 mm³	BR	1	Testing	N
St. Thomas Hospital, KCL (London, UK)	CMRA (T2-Prep)	0.55T Free Max	1.5 mm³	BR	2	Testing	N
St. Thomas Hospital, KCL (London, UK)	CMRA (T2-Prep)	0.55T Free Max	3.0 mm³	BR	2	Testing	N
Vall d′Hebron University Hospital (Barcelona, Spain)	CMRA (T2-Prep)	1.5T Sola	1.0 mm³	BR	5	Testing	Mix
St. Vincent’s Hospital (Sydney, Australia)	TWIST MRA	3T Prisma	1.09 × 1.09 × 1.19 mm	BR	1	Testing	N

This table shows the various MR datasets employed in the study. The first row represents the iT2PrepIR-BOOST dataset used for DL model training, while the other rows were used exclusively for model testing. For each dataset, the following information is shown: Origin (center and city), sequence, scanner type, (isotropic) spatial acquired resolution, image type, and patient information.

Data are presented as number (count) of datasets within each center.

*BL* black-blood, *BR* bright-blood, *CMRA* coronary magnetic resonance angiography, *MR* magnetic resonance, *MRA* magnetic resonance angiography, *T2-Prep* T2 preparation, *TWIST* time-resolved angiography with interleaved stochastic trajectories

### 2.4 Model(s) training

#### 2.4.1 Server specifics

A virtual environment (Anaconda) was created on a server running on Ubuntu 20.04.6 LTS, with an AMD Ryzen threadripper (AMD, Santa Clara, California) 3970 × 32-core processor × 64 processors, and one NVIDIA GeForce RTX 3090 (NVIDIA, Santa Clara, California) graphical processing unit (GPU). The following software packages were used in the virtual environment: Python (v. 3.9.18, Python Software Foundation, Delaware), PyTorch library (v. 2.0.1, Meta Platforms, Inc., Menlo Park, California), Computed Unified Device Architecture = 11.7, Computed Unified Device Architecture Deep Neural Network = 8.5.0, nnUNet (v. 2.2.1, Helmholtz Imaging / DKFZ, Heidelberg, Germany), and Hiddenlayer (v.0.2, GitHub repository: waleedka/hiddenlayer).

#### 2.4.2 Manual segmentation labels

GT segmentation was performed by a single operator (M.C.) using the “Medical Imaging Interaction Toolkit” (MITK v.2023.04). This consisted of a thresholding operation 26, 27, whereby the newly created mask (foreground) was assigned a value of “1”, while the background, a value of “0,” resulting in binary volumes. Label segmentation was performed on all N = 25 BR image sets and N = 15 BL image sets; the latter is lower due to the exclusion of low-quality images and time limitations due to the additional complexity of vessel wall segmentation. A total of 40 image sets were annotated (approximating 3950 slices) by delineating the region of interest slice by slice. Axial slices were used as the first choice, while the other orientations were used for border correction. Per patient, three masks were created: one for the BR dataset (“Lumen”, Supplementary Fig. S2) and two for the BL dataset (“Lumen” and “Outer Vessel Wall,” Supplementary Fig. S3). The vessel wall mask on the BL image sets was obtained by digital subtraction of the “Outer vessel wall” and the “Lumen,” using MITK. The BL segmentations were modified to assign a value of “0,” “1,” and “2” to the background, lumen, and vessel wall, respectively. In BL image sets, the aortic wall at the level of the aortic root (below the sinotubular junction) was not segmented due to difficulties in distinguishing it from surrounding tissues and delineating its border with the left ventricle.

#### 2.4.3 The “nnU-Net” protocol

The *BRnnUNet* was trained to automatically segment the aortic lumen on BR image sets, while the *BLnnUNet* to automatically segment the aortic lumen and vessel wall on BL image sets. A detailed description of the nnUNet can be found in the article by Isensee et al. [18] and on the corresponding GitHub repository (https://github.com/MIC-DKFZ/nnUNet).

The preprocessing step consisted of (1) cropping the 3D image sets to their non-zero regions, (2) generating the dataset fingerprints, and (3) designing the U-Net configurations. The dataset fingerprint included information about image size, shape, and physical spacing after the cropping operation, as well as voxel intensities. The nnUNet trainable configurations were the following: 2D, 3D low and full resolution, and 3D cascade. The model’s parameters included batch size, patch size, normalization scheme used, convolutions and pooling layers, amongst others (Supplementary Information, Tables S1 and S2 for *BRnnUNet* and *BLnnUNet*, respectively).

Training consisted of 5-fold cross-validation, with 1000 epochs per fold, each epoch being an iteration over 250 mini-batches. The initial learning rate was 0.01 and decreased according to the “Poly-Learning” function (Eq. (1)), while the employed nnUNet loss function was a combination of the dice loss (L_DICE_) and the cross-entropy loss (L_CE_).

(1) $$\mathrm{Learning Rate}={\left(1-\frac{\mathrm{epoch}}{{\mathrm{epoch}}_{\max}}\right)}^{0.9}$$

All configurations were trained for the *BRnnUNet* model, while only the 3D full resolution (found as the best-performing configuration for the *BRnnUNet)* was trained for the *BLnnUNet* model. Data were subdivided into training, validation, and testing with a 70:20:10% (17/25:5/25:3/25 datasets) split, respectively. To increase variability in the training data and minimize over-fitting, several data augmentation techniques were applied by nnUNet. These included rotation and scaling, Gaussian noise, Gaussian blur, brightness, contrast, simulation of low resolution, gamma augmentation, mirroring, binary operators, and removal of connected components. The last two were applied solely during the 3D cascade training [18].

**Fig. 2:**
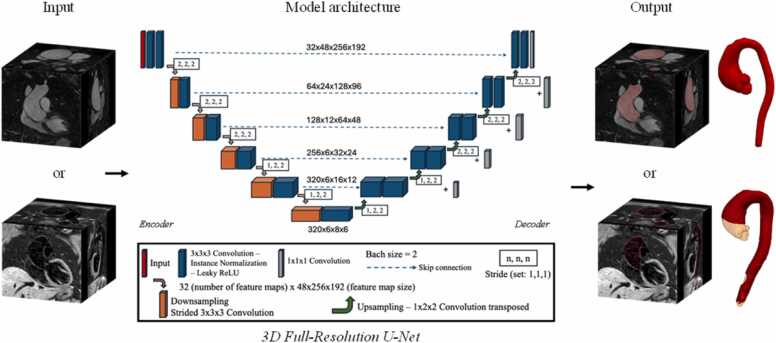
BRnnUNet and BLnnUNet architecture. Architecture of the 3D U-Net model used for training and inference. The input is a volumetric bright- or black-blood MRI scan, and the output is the corresponding segmented 3D mask. The U-Net architecture has the traditional encoder-decoder structure and relies on 3 × 3 × 3 convolutions, followed by instance normalization and leaky rectified linear units (Leaky ReLU). Downsampling (in the encoder) employs strided convolutions while upsampling (in the decoder) makes use of 1 × 2 × 2 transposed convolutions. The initial number of feature maps is set to 32 with size “48 × 256 × 192” and increases when going deeper into the neural network up to 320 feature maps with size “6 × 8 × 6.” Skip connections join layers of the encoder and decoder at the same level to maintain the high dimensionality of the data. The main difference between BRnnUNet and BLnnUNet lies in the feature map sizes at the network layers due to cropping in the preprocessing of the original data. The U-Net architecture, specifically represented in this image,e shows the BRnnUNet model. *3D* three-dimensional, *BRnnUNet* bright-blood nnUNet model trained to segment aortic lumen, *BLnnUNet* black-blood nnUNet model trained to segment aortic lumen and wall, *MRI* magnetic resonance imaging

The general U-Net architecture was a fixed parameter and the only difference between the *BR* and *BLnnUNet* models was the feature map size at the different layers. The U-Net architecture employed consisted of the typical encoder-decoder structure with skip connections, and 3 × 3 × 3 convolutions, followed by instance normalization and leaky rectified linear units. Downsampling (in the encoder) consisted of strided convolutions while upsampling (in the decoder) consisted of transposed convolutions (Fig. 2).

### 2.5 Inference and generalizability

The testing data were obtained from three different centers and included both healthy subjects and patients with aortic disease. Testing of the BRnnUNet model was done on 18 BR datasets, while testing of the BLnnUNet model was done on 14 datasets. The image contrasts included iT2PrepIR-BOOST, CMRA (T2-Prep) [24] with isotropic acquired resolutions ranging from 0.9 to 3.0 mm^3^ and a TWIST MRA dataset with acquired resolution of 1.09 × 1.09 × 1.19 mm. These image sets were obtained with different MRI scanners (Magnetom Sola, Aera, Prisma, and Free Max) at varying field strengths (0.55T, 1.5T, and 3T).

Two additional test datasets featuring pathological aortic conditions not represented in the training set and acquired with the iT2PrepIR-BOOST sequence were included for testing. These cases were excluded from the main results table due to their outlier status but are presented separately to illustrate the generalization capability and limitations of the proposed method. One case involved an aortic dissection, while the other featured a patient with a mechanical valve replacement and abnormal curvature of the descending aorta.

The GT labels for the testing datasets were generated by the same operator (M.C.) following the same process as for the GT labels of the training datasets (as described in Section 2.4.2).

GT and predicted segmentations were compared using the Dice similarity coefficient (DSC) (Eq. (2)) and Intersection over Union (IoU) (Eq. (3)). The DSC and IoU provide a value between 0 (no overlap) and 1 (perfect overlap), with values above 0.80 considered as good agreement.

(2) $$\mathrm{DSC}=\frac{2*|A\cap B|}{\left|A\right|+|B|}=\frac{2*\mathrm{TP}}{\left(\mathrm{TP}+\mathrm{FP}\right)+(\mathrm{TP}+\mathrm{FN})}$$

(3) $$\mathrm{IoU}=\;\frac{|A\cap B|}{|A\cup B|}=\frac{\mathrm{TP}}{\mathrm{TP}+\mathrm{FP}+\mathrm{TN}\;}$$

### 2.6 Postprocessing: extracting clinically relevant information

The software 3D Slicer (https://www.slicer.org/) [28] was employed for the postprocessing steps. Centreline tracking and (max) diameter measurements were performed using the “Extract Centreline” and “Cross-Section Analysis” modules, respectively, of the “Vascular Modelling Toolkit (VMTK)” extension (https://github.com/vmtk/SlicerExtension-VMTK). First, the start- and end-points of the vessel were detected, and then the centreline was extracted. The (max) diameter and CSA were measured at every point along the vessel orthogonal to the centreline. CPR was performed using the “Curved Planar Reformat” (CPR) module included in the “Slicer Sandbox” extension from https://github.com/PerkLab/SlicerSandbox. Using the previously extracted centreline, the 3D volume was reformatted along the course of the aorta. Two types of CPRs were performed: straightening and stretching [29], which allow the detection of diameter changes along the vessel’s length. Both reformats maintained isometry. The stretched reformat displays the vessel in a curved plane, thus showing the aorta structure in its entirety including the surrounding tissues and organs [29].

### 2.7 Qualitative analysis and statistics

All GT segmentations (used for model training) were qualitatively assessed by two independent readers: J.N. (cardiologist with 4 years of CMR experience) and S.L. (cardiologist, with 2 years of CMR experience). The level of agreement between the readers was assessed with the Cohen’s Kappa Test on SPSS (v. 27, IBM Corp., Armonk, New York).

Most of the predicted segmentations were qualitatively assessed by J.N. The scoring criteria used were proposed by Hepp et al. [16]:

1. Accurate segmentation without visually relevant errors (*No Errors*).
2. Minor segmentation errors at the start and/or end of the aorta and/or associated with branches (*Category I*).
3. Additional smaller irregularities of the segmented region due to additional or missing areas (*Category II*).
4. Larger irregularities, but aorta detected as a whole (*Category III*).
5. Larger parts of the aorta are not detected correctly (*Category IV*).

## 3 Results

### 3.1 BRnnUNet model: aortic lumen segmentation on bright-blood 3D volumes

#### 3.1.1 Qualitative assessment of ground truth

Qualitative assessment results for the GT segmentations are shown in Supplementary Table S3. The majority (94%) of the masks were scored as “error-free” or with “minor error” (24/26 and 25/26 for experts 1 and 2, respectively). The segmentations with “minor-error” classified as “2 – Category I” (10/26 and 14/26, respectively) had minor inclusion of voxels belonging to the origin of the celia plexus, right coronary artery (RCA), supra-aortic vessels, renal artery branches, coronary ostia, or in certain cases, the abdominal aortic branches. Additional small errors (“3 – Category II”) were found on 2/26 and 1/26 masks (experts 1 and 2, respectively). These segmentations had additional smaller irregularities which included the origin of supra-aortic vessels, left coronary artery or, in certain cases, abdominal aortic branches. The Cohen’s Kappa test resulted in a fair level of agreement (κ = 0.45, p = 0.006) on the qualitative score between the two independent readers. These errors were not corrected before DL training.

#### 3.1.2 U-Net model configuration(s)

The 3D full-resolution U-Net was the best-performing configuration for the *BRnnUNet* model, as tested on the validation dataset (DSC = 0.96) (Supplementary, Fig. S4). Postprocessing by nnUNet, which consisted of removing everything except the largest mask component (i.e., the aortic lumen), increased the DSC to 0.97.

#### 3.1.3 Inference on test datasets

The 3D *BRnnUNet* model was tested on N = 18 subjects, from seven different datasets obtained from centers in the United Kingdom, Spain, and Australia. Corresponding predictive measures are summarized in Table 2. The model (trained and) tested on iT2PrepIR-BOOST (1.5T Aera, 1.3 mm^3^) image sets resulted in a DSC of 0.97 ± 0.01 and IoU of 0.94 ± 0.01. The predicted segmentations on the other datasets achieved similar results (DSC = 0.89–0.97, IoU = 0.82–94) except for CMRA at 0.55T with an isotropic acquired resolution of 3 mm^3^. A decrease in the scores was observed for the low-resolution CMRA sequence (London, 0.55T Free Max, 3 mm^3^) on which the model obtained a DSC and IoU of 0.68 ± 0.23 and 0.52 ± 0.26, respectively. The 3D BRnnUNet was tested on an extra TWIST MRA dataset (CE, Sydney, 3T Magnetom Prisma, 1.09 × 1.09 × 1.19 mm) on which it obtained a DSC of 0.90 and IoU of 0.82 (Supplementary Fig. S5).

**Table 2:** Predictive measures of the *BRnnUNet* model on the test datasets.

Center (city, country)	Sequence	Scanner	Acquired resolution	Aortic disease (Y/N/Mix)	N	DSC (mean±SD)	IoU (mean±SD)
St. Thomas Hospital, KCL (London, UK)	iT2PrepIR-BOOST	1.5T Aera	1.3 mm³	Y	3	0.97±0.01	0.94±0.01
St. Thomas Hospital, KCL (London, UK)	iT2PrepIR-BOOST	1.5T Sola	1.8 mm³	N	4	0.96±0.02	0.92±0.03
St. Thomas Hospital, KCL (London, UK)	CMRA (T2-Prep)	1.5T Sola	0.9 mm³	N	1	0.97	0.94
St. Thomas Hospital, KCL (London, UK)	CMRA (T2-Prep)	0.55T Free Max	1.5 mm³	N	2	0.93±0.01	0.88±0.02
St. Thomas Hospital, KCL (London, UK)	CMRA (T2-Prep)	0.55T Free Max	3.0 mm³	N	2	0.68±0.23	0.52±0.26
Vall d′Hebron University Hospital (Barcelona, Spain)	CMRA (T2-Prep)	1.5T Sola	1.0 mm³	Mix	5	0.89±0.14	0.82±0.20
St. Vincent’s Hospital (Sydney, Australia)	TWIST MRA	3T Prisma	1.09 × 1.09 × 1.19 mm	N	1	0.90	0.82

The first row is the iT2PrepIR-BOOST dataset that was employed in model training. All the others were used exclusively for testing. The two reported predictive measures are: Dice similarity coefficient (DSC), and intersection over union (IoU).

Data are presented as number of cases (N) or mean ± standard deviation, as appropriate.

*BRnnUNet* bright-blood nnUNet model trained to segment aortic lumen, *CMRA* coronary magnetic resonance angiography, *MRA* magnetic resonance angiography, *SD* standard deviation, *T2-Prep* T2 preparation, *TWIST* time-resolved angiography with interleaved stochastic trajectories

#### 3.1.4 Visual representation of predicted segmentations

**Fig. 3:**
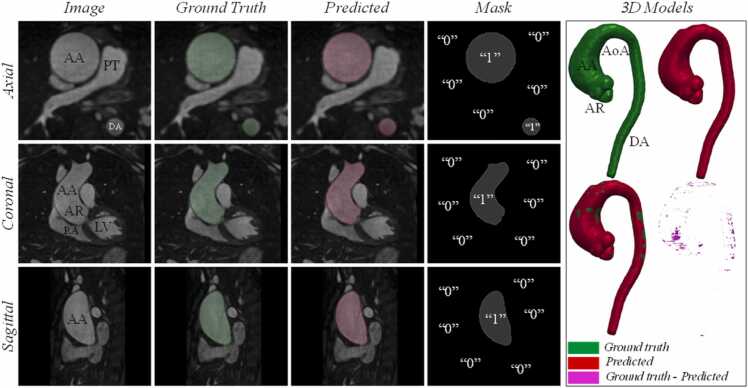
BRnnUNet predicted aortic lumen segmentation—iT2PrepIR-BOOST (1.5T Aera, 1.3 mm^3^) test dataset. Comparison of ground truth against predicted segmentations for patient n.32 with ascending aortic aneurysm acquired with iT2Prep-BOOST sequence (1.5T Aera, 1.3 mm^3^). Axial, coronal, and sagittal slices of bright-blood dataset showing the thoracic aorta. Light green: ground truth lumen segmentation, light red: predicted lumen segmentation. 3D models showing the ground truth (green), predicted (red), overlayed (green and red), and difference (ground truth – predicted) (magenta). The DSC = 0.97 and IoU = 0.95 suggest near-perfect agreement between manual and automatic segmentations. *3D* three-dimensional, *AA* ascending aorta, *AoA* aortic Arch, *AR* aortic root, *BRnnUNet* bright-blood nnUNet model trained to segment aortic lumen, *DA* descending aorta, *DSC* dice similarity coefficient, *IoU* intersection over union, *LV* left ventricle, *PT* pulmonary trunk, *RA* right atrium

**Fig. 4:**
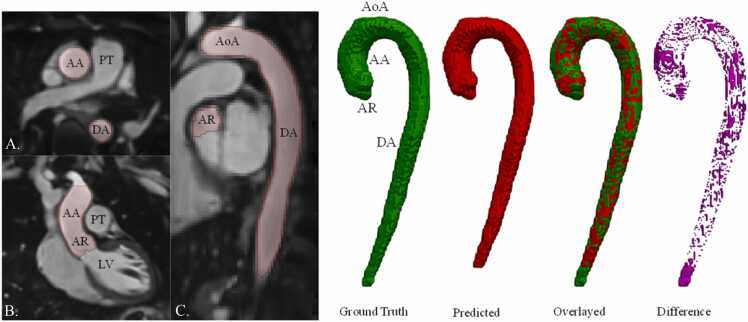
BRnnUNet predicted aortic lumen segmentation—iT2PrepIR-BOOST (1.5T Aera, 1.8 mm^3^) test dataset. Predicted aortic lumen segmentation on axial (A), coronal (B), and sagittal (C) slices. Three-dimensional (3D) models showing the ground truth (green), predicted segmentation (red), overlay (green and red), and difference (magenta) mask. DSC = 0,95, IoU =0.90. *AA* ascending aorta, *AoA* aortic Arch, *AR* aortic root, *BRnnUNet* bright-blood nnUNet model trained to segment aortic lumen, *DA* descending aorta, *DSC* dice similarity coefficient, *IoU* intersection over union, *LV* left ventricle, *PT* pulmonary trunk, *RA* right atrium

**Fig. 5:**
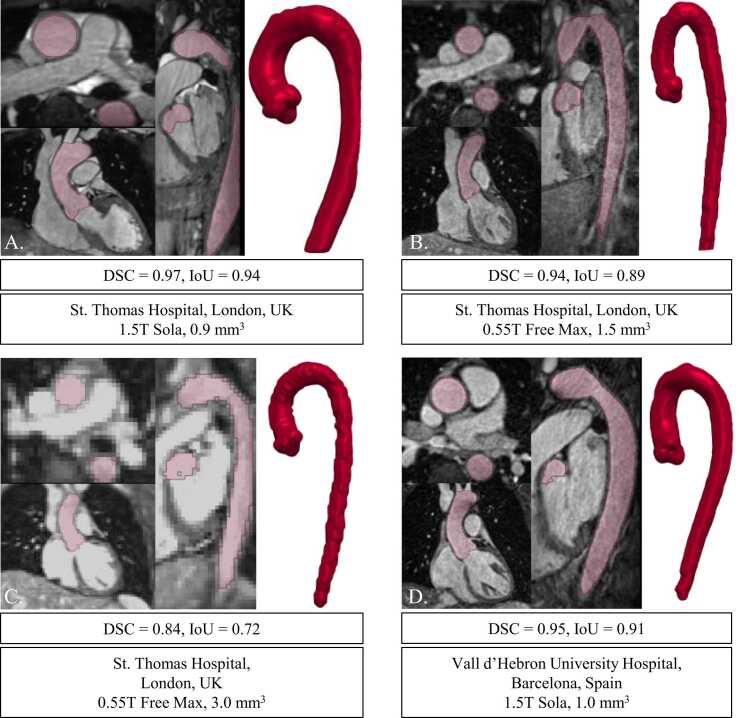
Inference of BRnnUNet on CMRA (T2-Prep) test datasets. Inference results of BRnnUNet on images from the four CMRA datasets used for testing. (A) CMRA (T2-Prep) from London, UK (1.5T Sola, 0.9 mm^3^), (B) CMRA (T2-Prep) from London, UK (low field 0.55T Free Max, 1.5 mm^3^), (C) CMRA (T2-Prep) from London, UK (low field 0.55T Free Max, 3.0 mm^3^), (D) CMRA (T2-Prep) from Barcelona, Spain (1.5T Sola, 1.0 mm^3^). *BRnnUNet* bright-blood nnUNet model trained to segment aortic lumen, *CMRA* coronary magnetic resonance angiography, *DSC* Dice similarity coefficient, *IoU* intersection over union, *T2-Prep* T2 preparation

Examples of *BRnnUNet* automatic segmentations are illustrated in Fig. 3, Fig. 4, Fig. 5, showing the GT and predicted segmentations. The GT and predicted segmentations for a patient with ascending AA, characterized by significant enlargement of the lumen, acquired with iT2PrepIR-BOOST (1.5T Aera, 1.3 mm^3^), are shown in Fig. 3. The predicted segmentation showed excellent agreement with GT, with a DSC and IoU of 0.97 and 0.95, respectively. The model achieved accurate segmentation of the aneurysm and aortic root, with noteworthy separation between the root lumen and the left ventricular outflow tract (LVOT), as shown in Supplementary Fig. S6.

The predicted segmentation on a dataset acquired with the iT2PrepIR-BOOST sequence (1.5T Sola) at a lower acquired resolution (1.8 mm^3^) is depicted in Fig. 4. Fig. 5 depicts the predicted segmentations on four different CMRA datasets (London, 1.5T Sola, 0.9 mm^3^; London, 0.55T Free Max, 1.5 mm^3^; London, 0.55T Free Max, 3 mm^3^; Barcelona, 1.5T Sola, 1 mm^3^). The model failed to properly detect and segment parts of the aorta on 2 out of 17 datasets (11%), 1 belonging to the CMRA dataset from Barcelona, Spain (1.5T, 1.0 mm^3^) and 1 from the CMRA dataset from London, UK (0.55T, 3.0 mm^3^).

#### 3.1.5 Qualitative assessment of predicted segmentations

All 17 (100%) testing datasets were scored as “error-free” (1 – No errors) or with “minor errors” (2 – Category I errors). All iT2PrepIR-BOOST datasets (1.5T Sola, 1.8 mm^3^ and 1.5T Aera, 1.5 mm^3^) were scored as error-free, and the minor involvement of aortic branches origin was deemed of no clinical significance. Out of five CMRA datasets at 1.5T (Sola, 0.9 mm^3^), two were classified as “2 – Category I errors” due to more appreciable involvement of the coronary artery ostia, in particular the RCA, in one case, and involvement of the celia artery, in the other.

#### 3.1.6 Computational efficiency

Model training with the use of one *NVIDIA 3090 GeForce RTX* GPU took 331 h (∼14 days) for all configurations and 86 h (≈ 3.5 days) for the 3D full-resolution configuration alone (5000 epochs, ∼63 s per epoch). Manual segmentation of one 3D dataset took approximately 2 h, while automatic segmentation (inference) of one 3D dataset with *BRnnUNet* was completed in ≈ 50 s, approximately ≈ 144 times faster.

### 3.2 BLnnUNet model: aortic lumen and wall segmentation on black-blood 3D volumes

#### 3.2.1 Qualitative assessment of ground truth

Qualitative assessment results for the GT segmentations are shown in Supplementary Table S4. The majority (100% and 92%) of GT masks were scored as error-free or with “minor error” (20/20 for both experts for lumen segmentation, and 14/14 and 12/14 for wall segmentation for experts 1 and 2, respectively). Additional small errors (“3 – Category II”) were found on 2/14 masks (expert 2) for wall segmentation. Lumen segmentation errors classified as “2 – Category I” (2/20 and 11/20 for experts 1 and 2, respectively) included minor involvement of the origin of the coronary, supra-aortic, or celia branches, or minor errors at the level of the aortic arch and root. On the other hand, vessel wall segmentations were scored as “2” (5/14 and 11/14, respectively) either due to the involvement of surrounding tissue or to early abdominal aorta not being segmented in some slices. The two datasets that obtained a “3” for the wall segmentations included part of the main pulmonary artery or other significant irregularities. The Cohen’s kappa test between the two readers resulted in a slight, but not significant, level of agreement for both the lumen (κ = 0.17, p = 0.18) and the outer vessel wall qualitative score on the segmentations (κ = 0.15, p = 0.18).

#### 3.2.2 Inference on test datasets

The *BLnnUNet* model was tested on two different BL datasets, the iT2PrepIR-BOOST (1.5T Aera) at the spatial acquired resolution of 1.3 mm^3^ (N = 8 for lumen and N = 2 for wall), and the iT2PrepIR-BOOST (1.5T Sola) at 1.8 mm^3^ (N = 2 for lumen and N = 2 for wall). Corresponding DSC and IoU are summarized in Table 3. The DSC and IoU for the lumen segmentation at 1.3 mm^3^ were equal to 0.95 ± 0.02 and 0.90 ± 0.03, respectively. For the vessel wall automated segmentations, the DSC was equal to 0.80 ± 0.0 and the IoU to 0.67 ± 0.02. The cross-resolution test on the iT2Prep-BOOST data (1.5T Sola, 1.8 mm^3^) resulted in a DSC of 0.96 ± 0.01 and IoU of 0.93 ± 0.02 for lumen segmentations, and a DSC of 0.82 ± 0.02 and IoU of 0.69 ± 0.02 for vessel wall masks.

**Table 3:** Predictive measures of the BLnnUNet model on the test datasets.

Center (city, country)	Sequence	Scanner	Acquired resolution	Aortic disease (Y/N/Mix)	N	DSC (mean±SD)	IoU (mean±SD)
*Lumen*							
St. Thomas Hospital, KCL (London, UK)	iT2PrepIR-BOOST	1.5T Aera	1.3 mm³	Y	8	0.95±0.02	0.90±0.03
St. Thomas Hospital, KCL (London, UK)	iT2PrepIR-BOOST	1.5T Sola	1.8 mm³	N	2	0.96±0.01	0.93±0.02

*Wall*							
St. Thomas Hospital, KCL (London, UK)	iT2PrepIR-BOOST	1.5T Aera	1.3 mm³	Y	2	0.80±0.01	0.67±0.02
St. Thomas Hospital, KCL (London, UK)	iT2PrepIR-BOOST	1.5T Sola	1.8 mm³	N	2	0.82±0.02	0.70±0.02

The first row for the lumen and wall sections of this table belongs to the iT2PrepIR-BOOST datasets which were employed for model training.

Data are presented as number of cases (N) or mean ± standard deviation, as appropriate.

*BRnnUNet* bright-blood nnUNet model trained to segment aortic lumen, *DSC* Dice similarity coefficient, *IoU* intersection over union, *SD* standard deviation

#### 3.2.3 Visual representation of predicted segmentations

**Fig. 6:**
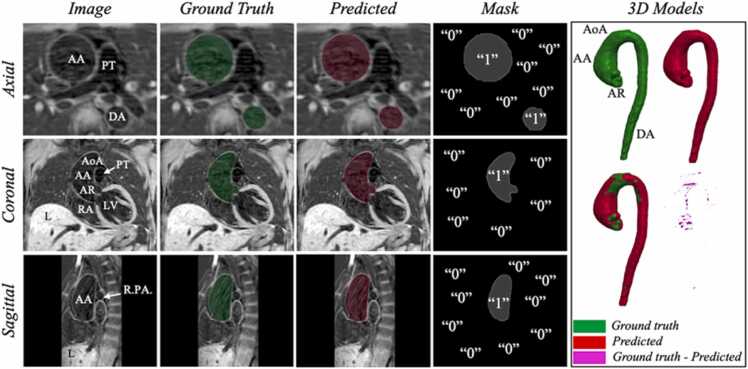
BLnnUNet predicted aortic lumen segmentation—iT2PrepIR-BOOST (1.5T Aera, 1.3 mm^3^) dataset. Inference result of BLnnUNet on one iT2Prep-BOOST (1.5T Aera, 1.3 mm3) black-blood dataset. Axial, coronal, and sagittal slices with ground truth and predicted segmentations. Right: 3D models showing the ground truth, predicted, overlayed, and difference. DSC = 0.97, IoU = 0.95. *3D* three-dimensional, *AA* ascending aorta, *AoA* aortic Arch, *AR* aortic root, *BRnnUNet* bright-blood nnUNet model trained to segment aortic lumen, *DA* descending aorta, *DSC* dice similarity coefficient, *IoU* intersection over union, *LV* left ventricle, *PT* pulmonary trunk, *RA* right atrium, *RPA* right pulmonary artery

**Fig. 7:**
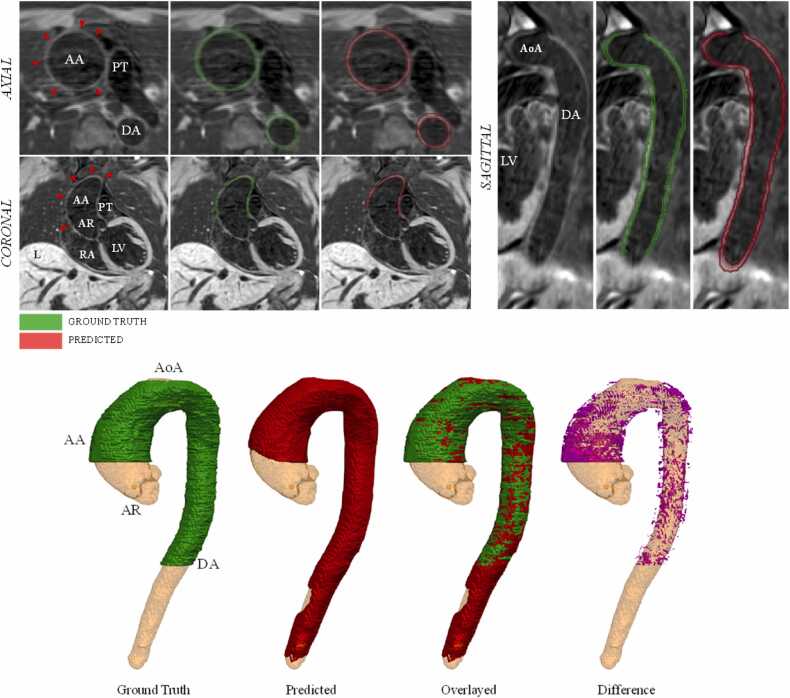
BLnnUNet predicted aortic wall segmentation—iT2PrepIR-BOOST (1.5T Aera, 1.3 mm^3^). iT2Prep-BOOST (1.5T Aera, 1.3 mm^3^). Axial, coronal, and sagittal slices with ground truth and predicted segmentation of the aortic wall. Bottom: 3D representation of aortic wall. Light yellow = predicted 3D segmentation of aortic lumen as a reference structure. DSC = 0.79, IoU = 0.6. Red arrowhead: aortic wall. *3D* three-dimensional, *AA* ascending aorta, *AoA* aortic Arch, *AR* aortic root, *BRnnUNet* bright-blood nnUNet model trained to segment aortic lumen, *DA* descending aorta, *DSC* dice similarity coefficient, *IoU* intersection over union, *LV* left ventricle, *PT* pulmonary trunk, *RA* right atrium

**Fig. 8:**
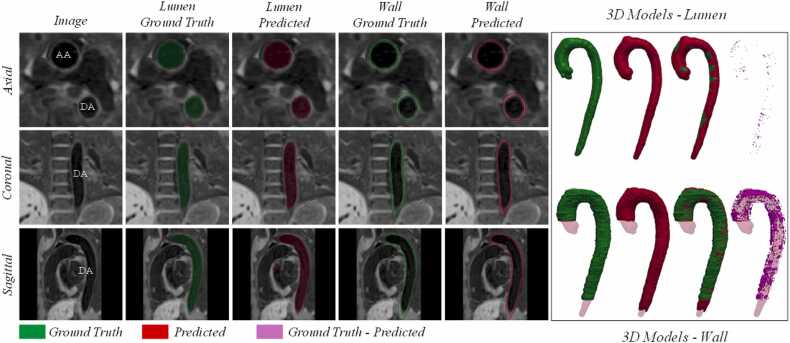
BLnnUNet aortic lumen and wall predicted segmentations—iT2PrepIR-BOOST (1.5T Sola, 1.8 mm^3^). Ground truth vs predicted segmentation of aortic lumen and wall by BLnnUNet on axial, coronal, and sagittal slices. Right: 3D models showing ground truth (green), predicted (red), overlayed (green and red), and difference (magenta) both for the lumen and the vessel wall. *3D* three-dimensional, *AA* ascending aorta, *BRnnUNet* bright-blood nnUNet model trained to segment aortic lumen, *DA* descending aorta

Examples of a 3D BL iT2PrepIR-BOOST (1.5T Aera, 1.3 mm^3^) dataset segmented by the BLnnUNet model are shown in Figs. 6 and 7 for the lumen and wall segmentation, respectively. The predicted mask of the aortic lumen was compared against the GT with a DSC of 0.97 and IoU of 0.95. The 3D volume shows an accurate representation of the lumen morphology, including the visible aneurysm at the level of the ascending aorta, and of the aortic root (Fig. 6). The predicted aortic wall mask compared to the GT resulted in a DSC and IoU of 0.79 and 0.65, respectively (Fig. 7). The 3D vessel wall predicted segmentation included a part of the descending aorta that was not part of the GT as deemed too difficult to distinguish from surrounding tissue. An example from one dataset belonging to the iT2PrepIR-BOOST (1.5T Sola, 1.8 mm^3^) is shown in Fig. 8. The BLnnUNet segmented the aortic lumen on BL image sets with good accuracy (DSC = 0.96, IoU = 0.92). The same was observed for the aortic wall, with a lower DSC of 0.83 and IoU of 0.71. It is important to note that the vessel wall of the aortic root was not segmented by the DL BLnnUNet model as it was not trained to do so.

Supplementary Figs. S7-S9 illustrate three examples of segmentation attempts on three particular cases: a Marfan’s syndrome patient who previously had undergone mechanical valve replacement and presented an abnormal curvature of the descending aorta (Supplementary Fig. S7), an aortic dissection case (Supplementary Fig. S8), and a patient with TAA (Supplementary Fig. S9).

#### 3.2.4 Qualitative assessment of predicted segmentations

Qualitative assessment of the predicted segmentations by the BLnnUNet model was performed by the expert J.N. Out of the four datasets scored, three were scored as “1 – No errors,” both for the lumen and vessel wall. One dataset was scored a “2 – Category I error” for the lumen segmentation due to a segment missing at the distal aortic arch, and “3 – Category II error” for the vessel wall due to segments missing at the root and the distal aortic arch.

#### 3.2.5 Computational efficiency

Overall, training of the 3D BLnnUNet model took approximately 90 h (3.8 days), and inference on a 3D BL iT2Prep-BOOST test dataset was performed in ≈ 50 s. Compared to the time taken to manually segment the lumen and the vessel wall on the same dataset, of ≈ 4 h, this was approximately ∼288 times faster.

### 3.3 Postprocessing: extracting clinically relevant information

#### 3.3.1 Centreline extraction and (max) diameter measurement

**Fig. 9:**
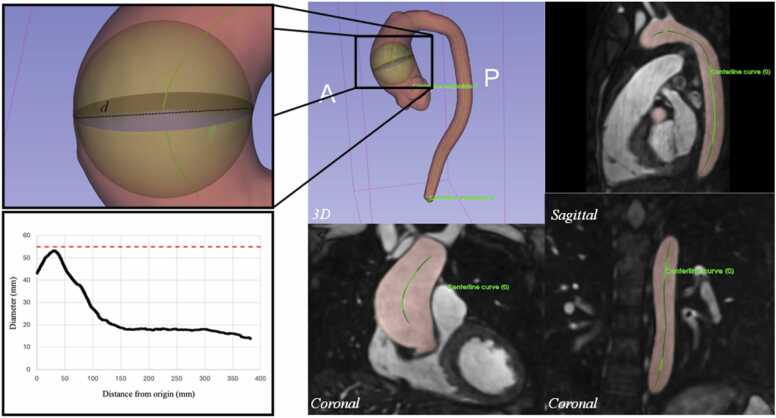
Outcome of (automatic) centreline extraction and (max) diameter measurement with 3D Slicer. Right: the extracted centreline (light green) is shown in the 3D model as well as in the sagittal and coronal slices. Top left: the aortic diameter (black dotted line) and cross-sectional area (purple disc/circle) can be measured at any point along the vessel orthogonal to the centreline. Bottom left: a plot of diameter (mm) against distance from the origin (mm) can be plotted for any 3D model to analyze diameter changes along the vessel’s length. Red dotted line: surgical threshold of 5.5 cm. In this patient, we can observe an aneurysm (∼ 53 mm) at the level of the ascending aorta (∼ 35 mm from the origin). *3D* three-dimensional

The automatic detection of the aorta’s start- and end-point and subsequent tracking and extraction of the centreline took ∼5 s. The software was also useful to semi-automatically obtain the (maximum) aortic diameter for every point location along the aorta, measured orthogonal to the centreline. Fig. 9 depicts the maximum inscribed sphere and corresponding maximum diameter for a representative patient with ascending AA (d = 53.41 mm). Additionally, 3D Slicer automatically generates a plot of aortic diameter (mm) against distance from the origin (mm). In this specific case, the point of maximum diameter is located at ∼35–40 mm from the origin.

#### 3.3.2 Curved planar reformat

**Fig. 10:**
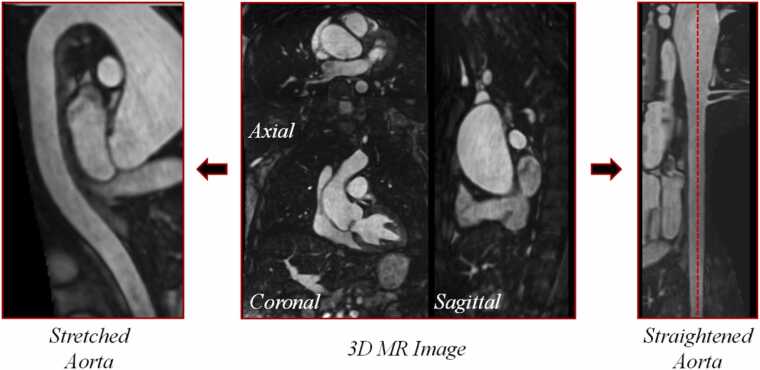
Outcome of (automatic) curved planar reformatting of the aorta. At the center of the image, we see the 3D MR image taken as an example of automatic CPR. On the left, there is the stretched aorta and, on the right, the straightened aorta. *3D* three-dimensional, *CPR* curved planar reformat, *MR* magnetic resonance

Starting with the original 3D volume, we utilized the previously extracted centreline to successfully implement and perform CPR on 3D Slicer. Two types of CPR were performed: straightening CPR, which obtained a straightened aorta, and stretching CPR, which obtained a curved “candy-cane” aorta (Fig. 10).

## 4 Discussion

The present study demonstrates that the two DL models (BRnnUNet and BLnnUNet) were able to generate a full 3D model of the aorta from 3D MR image sets in less than a minute. The BRnnUNet model showed excellent performance for the automatic segmentation of the aortic lumen on 3D BR image sets, with DSC above 0.95. The BLnnUNet model also showed excellent performance for aortic lumen segmentation on BL image sets, with an average DSC of 0.95, and vessel wall segmentation with an average DSC of 0.80. Automatic centreline extraction, diameter measurement, and CPR were successfully implemented based on the automatic segmentations using open-source software.

In this study, GT quality was overall good for both BR and BL datasets, with most of the masks scored as error-free or with minor errors of no clinical significance. The DL models have been shown to smooth out ramification errors at the level of aortic branches as predicted segmentations were scored generally better than GT. The qualitative scoring criteria used [16] leave space for subjectivity, which partly explains the moderate (for BR) and low agreement (for BL) between the readers.

The model’s performance was assessed by comparing GT and predicted masks with the use of DSC and IoU. In contrast to accuracy and specificity which can result in false high scores, these metrics penalize false positives [30]. The IoU results in lower values compared to DSC because of stronger penalization for under and over-segmentations [30]. The *BRnnUNet* model, although being trained on image sets from one dataset (iT2PrepIR-BOOST, 1.5T Aera, 1.3 mm^3^), has shown good performance when tested across different centers, sequences, acquired resolutions, and MR scanners. The same holds for the *BLnnUNet* model, which was tested across the same sequences but at two different acquired resolutions (1.3 and 1.8 mm^3^). The overall morphology of the aortic lumen was accurately represented by both models, despite the complex morphology and similar voxel intensity between the root lumen and the LVOT. The *BLnnUNet* segmentation of the aortic wall resulted in good overlap between GT and predicted masks, despite the low amount of training data and complexity of the task.

Both models performed well for different spatial acquired resolutions, scanner models, field strength, and contrast despite the small training data size. The difference in shapes and morphology of the aorta in the subjects with aortic disease increased the variability and heterogeneity of the training data, as compared to a more homogeneous healthy population. Second, the data augmentation steps applied by the “nnU-Net” further increased the variability in the training data.

The “nnU-Net” framework, compared to existing DL models, presents several advantages, including automatic adaptation to different datasets and minimal requirement of manual input 18, 19. Overall, the DSC for DL segmentation of the aortic lumen in previous studies varied between 0.85 and 0.95 [31], with the majority of research performed on computed tomography (CT) scans 11, 12, 13, 32, 33. One example is the research performed by Li et al. [27], which trained a 3D nnUNet algorithm on 88 CT datasets and obtained a DSC of 0.97. The study by Hepp et al. [16] trained a convolutional neural network on 70 3D aortic lumen datasets obtained with a non-CE MR sequence (resolution: 1.2 × 1.2 × 2.5 mm) of the thorax. The performance of their model on unseen data resulted in a DSC of 0.85 [16], despite the larger amount of training data compared to our study. Berhane et al. [15] developed a 3D U-Net with Dense Net-based blocks instead of convolutional layers, to segment the aortic lumen on 4D flow MRI, and obtained a DSC of 0.95 [15]. Garrido-Oliver et al. employed nnUNet for the automatic segmentation of the aortic lumen [34] on 4D flow phase-contrast MRA reconstructions and obtained a DSC of 0.95. The spatial resolution used was 2.5 mm^3^ isotropic [34]. Although several studies have utilized DL segmentation models for the aortic wall 35, 36, as far as we know, this is the first time the nnUNet has been employed for vessel wall segmentation on 3D MR image sets. The present study demonstrates the overall good performance of nnUNet both for aortic lumen and vessel wall segmentations, with similar, if not higher, DSC compared to existing literature, trained on fewer data and higher resolution 3D MR image sets.

The incorporation of centreline extraction into the postprocessing pipeline was motivated by the fact that the aortic diameter is often overestimated when the cross-section is not taken orthogonal to the centreline [6]. With advancements in technology and the development of 3D imaging techniques for both CT and MRI, automated centreline-based diameter measurement has become feasible, thus facilitating the accurate measurement of the aortic luminal diameter [5]. Three-dimensional measurements, such as lengths, volumes, and tortuosity, might be better clinical predictors of aortic adverse events (AAEs) compared to (max) diameter, as the latter fails to capture the 3D complexity of aortic growth 6, 37, 38, 39. Furthermore, more than half of patients have been shown to develop AAEs before reaching the surgical threshold of 5.5 cm 6, 37, 38, 39. As reducing the threshold would signify increasing the number of patients undergoing surgery risks, various predictive measures have been proposed [6], such as ascending aortic length 38, 39, aortic volume [38], aortic tortuosity (defined as the 3D centreline length divided by the straight-line distance between end-points) 37, 40, 41, and CSA/height index [38]. Having a 3D rendering of the aorta enables clinicians to accurately measure these parameters using a variety of 3D visualization software.

Automated medical image segmentation offers numerous advantages over traditional manual methods 10, 18. First, it drastically reduces the time required for image analysis, allowing for faster clinical decision-making 10, 18, as demonstrated here by the faster segmentation with the *BRnnUNet* and *BLnnUNet* models of 140 and 280 times compared to a human operator, respectively. Second, it reduces the variability associated with manual segmentation, providing consistent and reproducible results regardless of the operator’s experience 4, 10. Finally, the implementation of CPR into the pipeline allowed us to automatically perform straightening and stretching CPR with the previously extracted centreline as a reference, as opposed to the manual selection of multiple points along the vessel, which is time-intensive and prone to errors. Furthermore, it offers several advantages over commercial tools that mainly focus on vessel diameter measurement and centreline reformation. It enables measurement of complex parameters such as length, volume, tortuosity and potentially also 3D aortic modeling using advanced computational methods. Unlike 2D lumen-based analysis methods, the proposed network also segments the vessel wall, thus facilitating more detailed analysis. DL models are robust to noise and artifacts, ensuring standardized and reproducible results. Combined with iT2PrepIR-BOOST imaging, it allows comprehensive assessment from a single scan. GPU acceleration enables fast inference with minimal manual correction, making it a scalable, future-proof solution for aortic evaluation.

## 5 Limitations

The main limitation of this study was the limited amount of GT labels, as manual segmentation is labor-intensive and time-consuming, especially for 3D data. Additionally, the model was trained on data from a single center and a single sequence, and therefore training could be expanded to include other MRA sequences obtained from different centers, MR scanners, and resolutions, to increase heterogeneity in the data, and therefore generalizability. Another factor limiting inference was MR image quality, as the model decreased in performance on those scans with a higher degree of noise and/or artifacts. This can also be improved by expanding training data and refining data augmentation strategies. The lowest performance was observed on the low-field (0.55T) CMRA dataset (London, 3.0 mm^3^) and may be attributed to several factors. One is the use of a low-field scanner, which has a lower signal-to-noise ratio compared to the higher-field systems on which the DL models were trained, and second, the resolution is lower compared to the training samples. It is worth noting that in one of the two image sets from the same dataset, the model performed well (DSC = 0.84), suggesting that the image quality of the single scan may have played a role. More scans are required to understand whether the difference stems from the image itself or the generalizability limitations of the DL networks. Additionally, there is a need for further validation in a larger number of subjects, as well as investigating generalizability across different MR scanner vendors. Also, with stronger computational power, training and inference time could be decreased even further.

The performance of the models on pathological cases that were not adequately represented in the training datasets is also an important limitation of the current study. The models failed to accurately segment the aortic lumen in a patient with aortic dissection, nor were they able to differentiate between true and false lumens, as such functionality was beyond the scope of this study. In a patient who had undergone mechanical valve replacement, the models successfully segmented the aortic root, ascending aorta, and most of the remaining vessel but struggled to accurately segment a portion of the descending aorta characterized by an abnormal curvature of the vessel. This highlights the necessity of expanding the training dataset to include a wider variety of anatomical deviations, particularly those resulting from surgical interventions or severe pathological conditions. Finally, cases involving pleural effusion represent another important category warranting inclusion in future training datasets. On the other hand, the model demonstrated good performance in cases involving normal aortic shape, aortic dilatation, and aneurysms. The latter, although a pathology, does not typically lead to extreme deviations in aortic shape (other than size) and was well represented in the training data. Future research could include CE BR image sets in the training dataset to improve the model’s performance on CE aortic scans.

## 6 Conclusions

In summary, we demonstrate that nnUNet enables automatic segmentation of the aortic lumen (mean DSC of 0.95) and vessel wall (mean DSC of 0.8) on 3D aortic datasets with different contrasts, acquired resolution, scanner type, and field strength for a single vendor, despite a relatively small number of training datasets. Future research should focus on further validation of these models in larger and more diverse patient populations. Additionally, integrating these models with other imaging modalities and clinical data could enhance their predictive capabilities and broaden their clinical applicability.

## Funding

The authors acknowledge financial support from (1) King’s BHF Centre for Award Excellence RE/24/130035 and RG/20/1/34802, (2) 10.13039/501100000266EPSRC EP/V044087/1, (3) Wellcome EPSRC Centre for Medical Engineering (NS/A000049/1), (4) Millennium Institute for Intelligent Healthcare Engineering ICN2021_004, 10.13039/501100002850FONDECYT 1250261 and 1250252, (5) IMPACT, Center of Interventional Medicine for Precision and Advanced Cellular Therapy, Santiago, Chile. ANID—Basal funding for Scientific and Technological Center of Excellence, IMPACT, #FB210024, (6) the Department of Health through the 10.13039/501100000272National Institute for Health Research (NIHR) comprehensive Biomedical Research Centre award, (7) 10.13039/100006662NIHR Cardiovascular MedTech Co-operative and (8) the Technical University of Munich – Institute for Advanced Study. The views expressed are those of the authors and not necessarily those of the BHF, NHS, the NIHR, or the Department of Health. J.N. is supported by the Royal Australasian College of Physicians’ Bushell Travelling Fellowship & a European Association of Cardiovascular Imaging Research Grant.

## Author contributions

**Jim Pouliopoulos:** Resources, Writing – review & editing. **Simon J. Littlewood:** Writing – review & editing, Validation, Investigation. **Reza Hajhosseiny:** Resources, Writing – review & editing. **Matteo Cesario:** Writing – review & editing, Writing – original draft, Visualization, Validation, Software, Methodology, Investigation, Formal analysis, Data curation, Conceptualization. **René M. Botnar:** Supervision, Project administration, Funding acquisition, Conceptualization. **Thomas J. Fletcher:** Writing – review & editing, Supervision, Methodology. **Andrew Jabbour:** Resources, Writing – review & editing. **James Nadel:** Writing – review & editing, Validation. **Carlos Castillo-Passi:** Investigation. **Anastasia Fotaki:** Investigation. **Jose Rodriguez-Palomares:** Investigation. **Ruperto Olivero:** Investigation. **Claudia Prieto:** Writing – review & editing, Supervision. **M. Eline Kooi:** Writing – review & editing, Supervision.

## Declaration of competing interests

The authors declare that they have no known competing financial interests or personal relationships that could have appeared to influence the work reported in this paper.

## Data Availability

The current ethics do not allow for data sharing. Upon a reasonable request, software code(s) can be shared.

## Footnotes

Appendix A Supplementary data associated with this article can be found in the online version at doi:10.1016/j.jocmr.2025.101923.
