# Supplementary material for: Automated segmentation of thoracic aortic lumen and vessel wall on three-dimensional bright- and black-blood magnetic resonance imaging using nnU-Net
Source: J Cardiovasc Magn Reson. 2025 Jun 11;27(2):101923. doi: 10.1016/j.jocmr.2025.101923 (PMC12780295; doi:10.1016/j.jocmr.2025.101923)

**Supplemental Data.** *In the order they show up in the article.*

**Supplementary, Fig. S1.** (Colour)

**Title:** Range of (aortic) conditions from the thirty patients enrolled in the study by Munoz *et al.* [4]

**Description**: Aortic conditions are categorized based on the anatomical location – *aortic root*, *ascending aorta*, or *descending aorta*. A few patients had a more *general* classification of their aortic disease (General).

**Supplementary, Fig. S2.** (Colour)

**Title:** Ground truth Segmentation of aortic lumen on bright blood.

**Description:** Example of ground truth (light green) segmentation of the aortic lumen on a 3D bright blood MR image obtained with the iT2PrepIR-BOOST sequence. All three orientations – axial, coronal, and sagittal – are shown. Abbreviations: AA = Ascending Aorta, AoA = Aortic Arch, AR = Aortic Root, DA = Descending Aorta, LV = Left Ventricle, PT = Pulmonary Trunk, RA = Right Atrium.

**Supplementary, Fig. S3.** (Colour)

**Title:** Ground truth segmentation of aortic lumen and vessel wall on black blood.

**Description:** Example of ground truth (green) segmentation of the aortic lumen and vessel wall on a 3D black blood MR image obtained with the iT2PrepIR-BOOST sequence. All three orientations – axial, coronal, and sagittal – are shown. Abbreviations: AA = Ascending Aorta, AoA = Aortic Arch, DA = Descending Aorta, LV = Left Ventricle, PT = Pulmonary Trunk, V = Vertebral Column. Red arrowheads: Aortic wall.

**Supplementary Table S1.**

**Title:** 3D BRnnUNet model parameters.

**Description:** Parameters for Data Pre-processing, Normalization, and U-Net Architecture.

| DATA AND PREPROCESSING PARAMETERS | |
| --- | --- |
| Batch Size | 2 |
| Patch Size | 48x256x192 |
| Median image size in voxels | 96.0, 448.0, 342.0 |
| Spacing | 1.29, 0.88, 0.88 |
| NORMALIZATION PARAMETERS | |
| Normalization schemes | Z-Score Normalization |
| Mask used for normalization | False |
| U-NET ARCHITECTURE PARAMETERS | |
| U-Net Class Name | “PlainConvUNet” |
| Number of initial features | 32 |
| Number of convolutions per stage (encoder) | [2, 2, 2, 2, 2, 2] |
| Number of convolutions per stage (decoder) | [2, 2, 2, 2, 2] |
| Number of pooling operation per axis | [3, 5, 5] |
| Kernel size for pooling operation at each stage | [[1, 1, 1], [2, 2, 2], [2, 2, 2],  [2, 2, 2], [1, 2, 2], [1, 2, 2]] |
| Kernel size for convolution operation at each stage | [3, 3, 3] |
| Maximum number of features | 320 |

**Supplementary Table S2.**

**Title:** 3D BLnnUNet model parameters.

**Description:** Parameters for data pre-processing, normalization, and U-Net architecture.

| DATA AND PREPROCESSING PARAMETERS | |
| --- | --- |
| Batch Size | 2 |
| Patch Size | 56x224x192 |
| Median image size in voxels | 104.0, 448.0, 342.0 |
| Spacing | 1.29, 0.88, 0.88 |
| NORMALIZATION PARAMETERS | |
| Normalization schemes | Z-Score Normalization |
| Mask used for normalization | False |
| U-NET ARCHITECTURE PARAMETERS | |
| U-Net Class Name | “PlainConvUNet” |
| Number of initial features | 32 |
| Number of convolution per stage (encoder) | [2, 2, 2, 2, 2, 2] |
| Number of convolution per stage (decoder) | [2, 2, 2, 2, 2] |
| Number of pooling operation per axis | [3, 5, 5] |
| Kernel size for pooling operation at each stage | [[1, 1, 1], [2, 2, 2], [2, 2, 2],  [2, 2, 2], [1, 2, 2], [1, 2, 2]] |
| Kernel size for convolution operation at each stage | [3, 3, 3] |
| Maximum number of features | 320 |

**Supplementary, Table S3.**

*Table 2. Qualitative assessment of Ground Truth aortic lumen on bright blood – Scores by expert readers.*


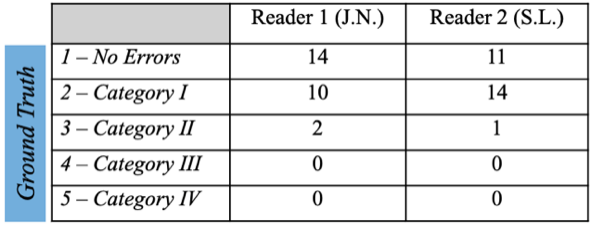


**Supplementary, Fig. S4.** (Colour)

**Title:** BRnnUNet – Best performing configuration on validation datasets.

**Description**: Results of “Find Best Configuration” on “nnU-Net” for the BRnnUNet model. The best-performing configuration with the highest Dice score is the 3D Full-Resolution (green bar). X-axis: Configurations, Y-axis: Dice-Score tested on the validation dataset.


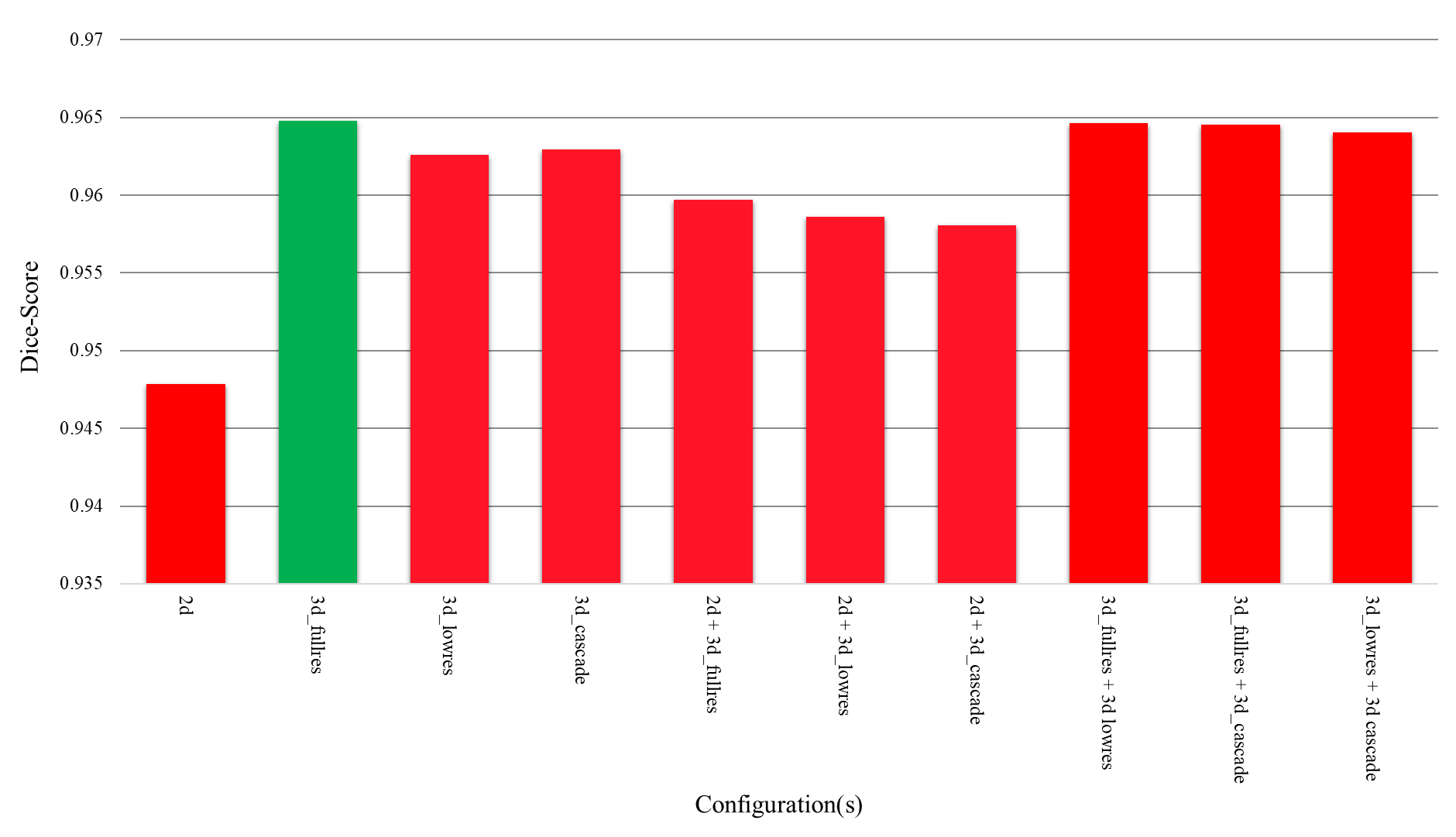


**Supplementary Fig. S5**

**Title:** BRnnUNet prediction of a contrast-enhanced aortic dataset – Twist MRA, 1.09x1.09x1.19 mm, St. Vincent’s Hospital, Sydney, Australia.

**Description:** The resulting Dice Similarity Coefficient (DSC) and Intersection over Union (IoU), when compared with the ground truth segmentations, were 0.90 and 0.82, respectively. The image below (Fig. C) illustrates the model’s segmentation performance across the three anatomical planes – axial, coronal, and sagittal – with the ground truth segmentation shown in green and the model’s prediction in red. Overall, except for the aortic root and the initial portion of the ascending aorta, the model accurately segmented the vessel. We attribute the observed decline in segmentation accuracy to three primary factors: (1) the use of different imaging sequences—iT2PrepIR-BOOST and TWIST MRA; (2) the presence of contrast medium; and (3) reduced image quality in the TWIST dataset compared to the original training data. Among these, the lower image quality—particularly at the aortic root—is likely the most significant contributor to the decreased performance, as illustrated by the provided image examples.


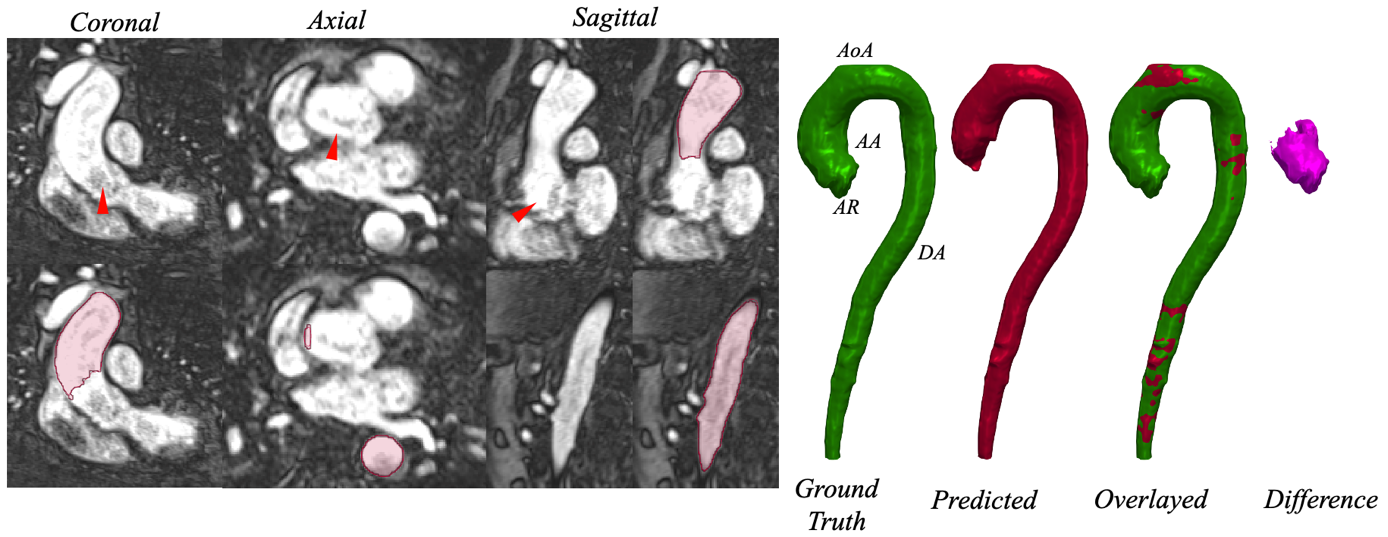


**Supplementary Fig. S6.** (Colour)

**Title:** BRnnUNet predicted segmentation of the aortic root on three datasets – iT2PrepIR-BOOST (1.5T Aera, 1.3 mm^3^)

**Description:** Comparison of ground truth and predicted segmentation of the aortic root for the three datasets from the iT2PrepIR-BOOST (1.5T Aera, 1.3 mm3). The aortic root was accurately segmented for pat. 18 and pat.32 by the BRnnUNet model, while for pat. 12 the model incorrectly included a part of the left ventricle outflow tract (LVOT).


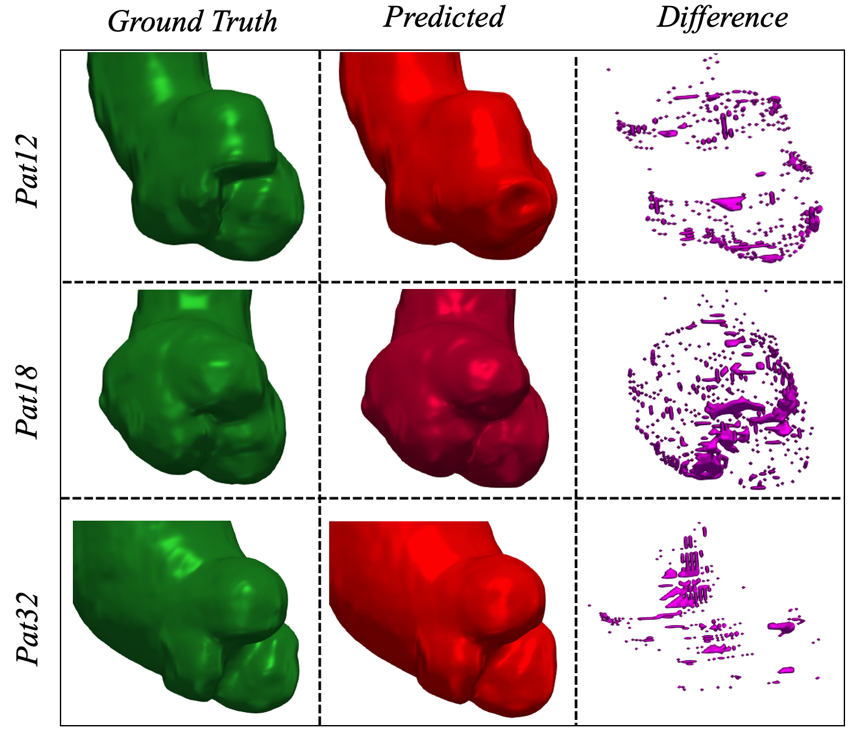


**Supplementary Table S4**

Table 4. *Qualitative assessment of Ground Truth aortic lumen and wall on black blood – Scores by expert readers.*


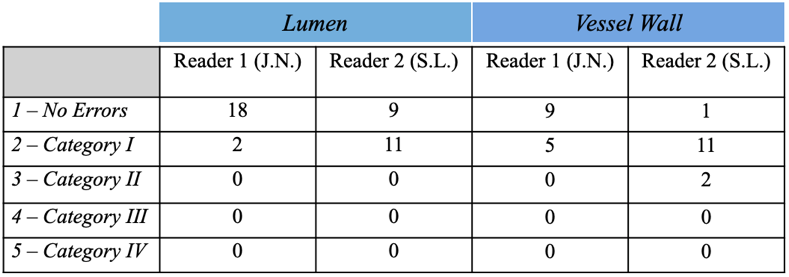


**Supplementary Figure S7.** (Colour)

**Title:** BRnnUNet and BLnnUNet predicted segmentation of the aorta in a Marfan’s syndrome patient that had undergone mechanical valve replacement – iT2PrepIR-BOOST (1.5T Aera, 1.3 mm^3^)

**Description:** Overall, the BRnnUNet model performed quite well on this dataset. The DSC was equal to 0.91 and the IoU to 0.84, indicating good agreement between the ground truth and automatic segmentation. The model accurately segmented most of the aorta, including the aortic root and ascending aorta. The main limitation to this specific case can be seen in a part of the descending aorta, characterized by an abnormal, curved shape, with great variation compared to a normal case. This amount of variation was not seen by the model during training, hence why this discrepancy between ground truth and predicted segmentations. A similar pattern can be seen in the black-blood images (DSC = 0.89, IoU = 0.80). Of note, the aortic wall was not segmented due to time constraints.


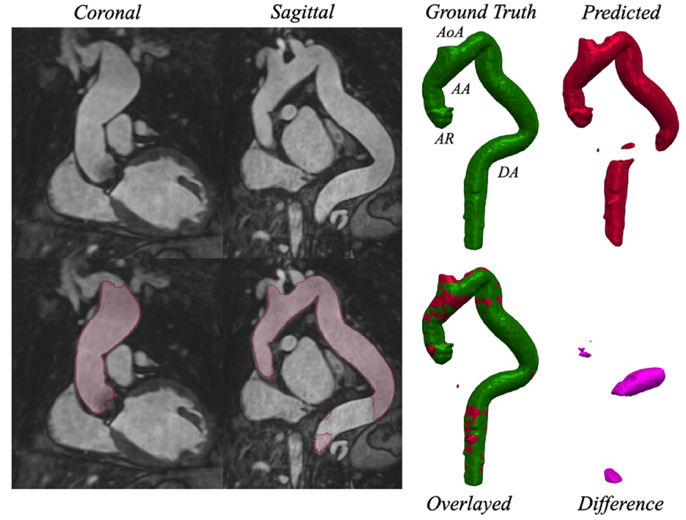

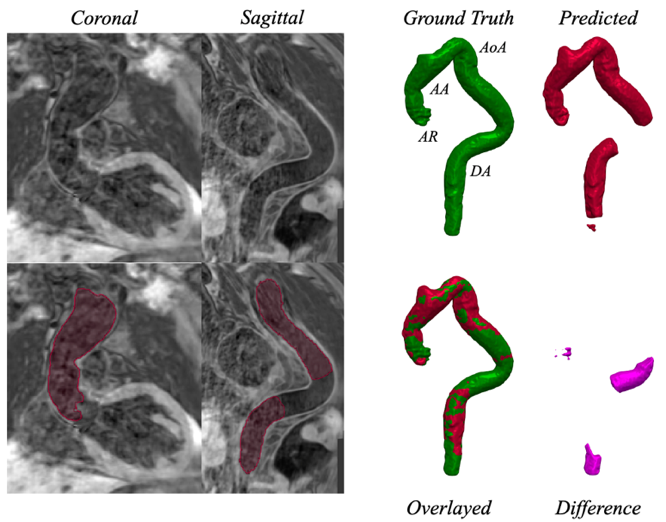


**Supplementary Figure S8.** (Colour)

**Title:** BRnnUNet and BLnnUNet predicted segmentation of the aorta in a patient with aortic dissection – iT2PrepIR-BOOST (1.5T Aera, 1.3 mm^3^).

**Description:** The BLnnUNet and BRnnUNet models struggled to accurately segment the aortic lumen across various regions of the aorta, particularly in areas affected by dissection. Notably, the segmentation was more successful at the level of the aortic root and ascending aorta, where the vessel was delineated correctly. These results are consistent with the model's limitations, as it was not trained to distinguish between true and false lumens.


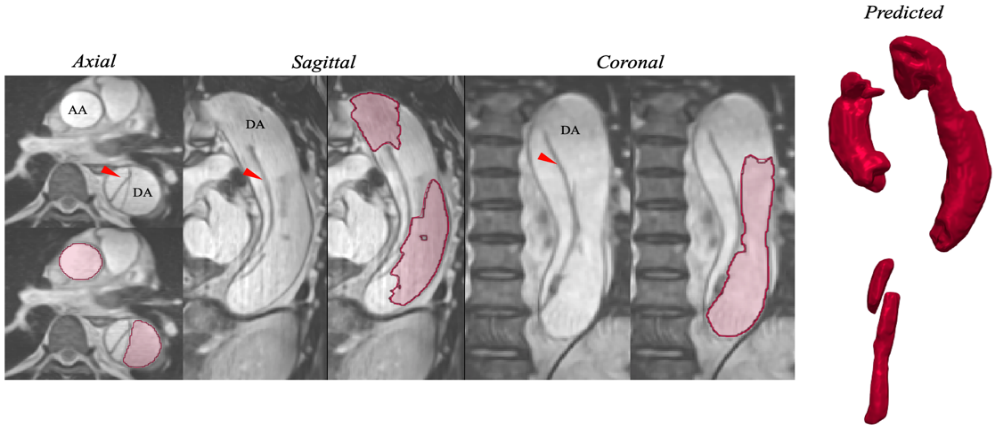

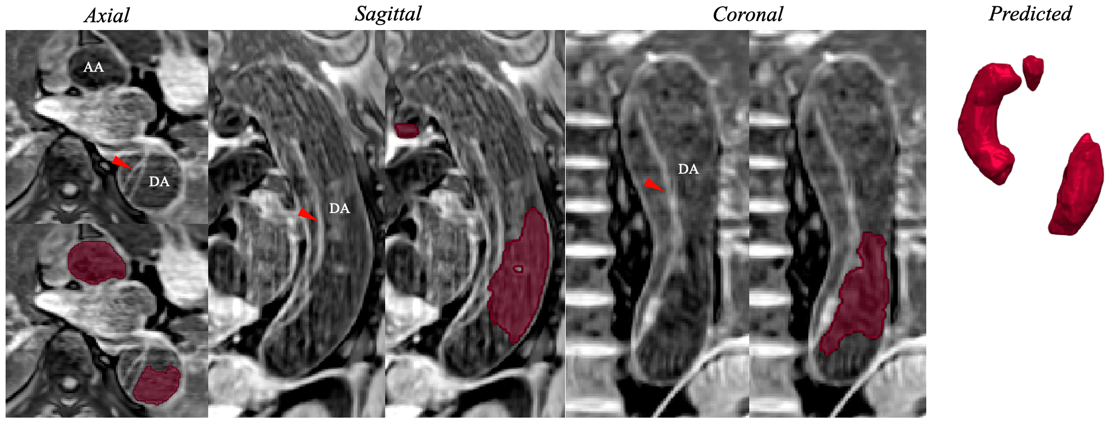


**Supplementary Fig. S9.** (Colour)

**Title:** BRnnUNet prediction of an aortic aneurysm case – CMRA (T2Prep), 1.0 mm^3^, Vall d’Hebron University Hospital (Barcelona, Spain).

**Description:** This dataset presents a patient with a thoracic aortic aneurysm, clearly visible in the ascending aorta. The model successfully performed segmentation of the full aorta with a DSC of 0.96 and IoU of 0.93.


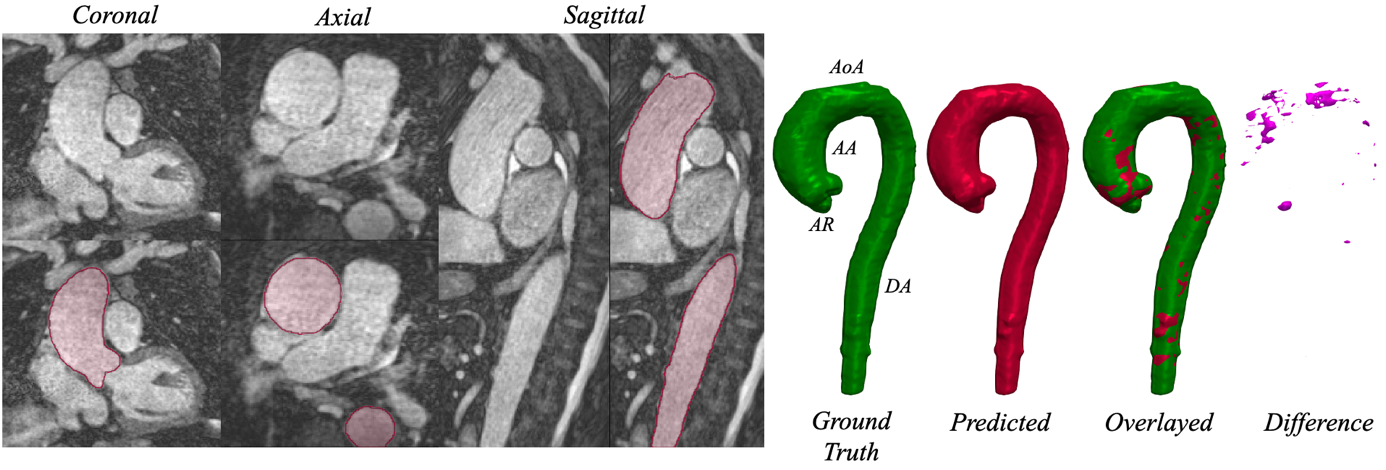

Supplement: Supplementary file 1 — Supplementary material [file mmc1.docx]
